# Religious Coping and Mental Adjustment to Cancer Among Polish Adolescents

**DOI:** 10.1007/s10943-023-01858-9

**Published:** 2023-07-05

**Authors:** Małgorzata M. Puchalska-Wasyl, Magdalena Małaj

**Affiliations:** 1 Department of Personality Psychology, Institute of Psychology, The John Paul II Catholic University of Lublin, Al. Racławickie 14, 20-950 Lublin, Poland; 2 TERPA, Lublin, Poland

**Keywords:** Religious coping, Mental adjustment, Support, Cancer, Adolescence

## Abstract

Adults suffering from chronic illnesses are more likely to look to God for support (positive religious coping; PRC) than to fight against God (negative religious coping; NRC). What about when cancer occurs during adolescence—a period of questioning the worldview and values, and at the same time searching for the sacred? Our study aimed to establish the relationships between PRC, NRC, and mental adjustment to cancer among youth and determine support’s role in these relationships. The study was conducted in Poland and included 88 adolescent cancer patients who completed the Brief RCOPE and the Mini-MAC. Additionally, general well-being and support were assessed. We found that PRC was positively related to constructive adjustment style (CAS), whereas NCR was related to destructive adjustment style (DAS). Adolescents with cancer were higher in PRC than in NRC and were higher in CAS than in DAS. In young women, CAS was higher than in men. Finally, at a level of received support rated as very high, PRC promoted fighting spirit and well-being.

## Introduction

According to the World Health Organization (WHO), deaths caused by cancer account for a considerable percentage of all deaths. More than 19 million new cancer cases were reported worldwide in 2020 alone, of which as many as 10 million were fatal (Sung et al., 2021). The predominant cancers among children and adolescents (aged 0–19 years) include leukemias, lymphomas, and brain tumors (Siegel et al., 2021). Adolescence is not only very important for physical, mental, and social development, but is also a crucial time for religious development—the formation of religious identity and its maintenance in later life (Desmond et al., 2010). Religious and spiritual phenomena, such as questioning the world view and values, looking for meaning and purpose, the desire to experience transcendence, and the search for the sacred, are considered as developmental tasks characteristic of this period (Levenson et al., 2013). During adolescence, young people revise religious knowledge previously transmitted by authority figures and significant others. Religious transformation is also facilitated by moral and intellectual doubts related to religion, and various types of crises that arise during this period. As a result, religious knowledge can be reformulated and adopted anew, but it can also be rejected (Talik, 2013). Breaking away from one's current belief system, morality and practices, as well as disconnecting from a religious or spiritual community—what is referred to in the literature as deconversion—is today seen by many scholars as a particularly important aspect of youth religious change (Puchalska-Wasyl et al., 2022; Zarzycka et al., 2022). If cancer occurs during adolescence, it is conceivable that a young person will turn away from God, abandon faith, and start looking for non-religious strategies to cope with this difficult situation. However, researchers point out that people suffering from chronic diseases more often turn to God with trust, seeking support in Him, than they fight with God (Koenig, 1994). It is consistent with research conducted among Polish general upper secondary school students (*N* = 451), which revealed that young people most often used religious coping strategies in liminal situations, such as illness. In addition, this way of coping was more effective in more religious adolescents (Talik, 2013).

### Positive and Negative Religious Coping

Kenneth I. Pargament, the author of the religious coping theory, defined religion as “a search for significance in ways related to the sacred” (Pargament, 1997, p. 32). In his opinion, religion provides a person with the necessary help resources, whose aim is to maintain meanings in a situation of stress or to transform them if they prove impossible to maintain (Pargament, 1996). The basic functions of religious coping in difficult situations include looking for meaning in life, achieving greater mastery over and control of one’s life, getting comfort from God, attaining closeness with God and with other people, and seeking a transformation in one’s life. Religious coping may be positive or negative. Positive religious coping (PRC) is associated with the experience of a secure attachment to the sacred and involves adopting a benevolent world view. Negative religious coping (NRC), in contrast, places emphasis on spiritual tension and conflicts with the sacred that manifest themselves in struggling with God and others (Pargament et al., 2011).

### Religious Coping in the Context of Illness and Health Status

In their review of research and meta-analyses, Mueller et al. (2001) concluded that most studies found patients’ religious involvement and spirituality to be related to better health outcomes, including longevity, coping skills, and health-related quality of life (even during terminal illness), and to less depression, anxiety, and suicide. Kaliampos and Roussi (2017) found that religious coping of cancer patients (*N* = 86) during chemotherapy was a predictor of their positive affect seven months later. Other research including patients with advanced cancer (*N* = 170) showed that health-related quality of life was positively associated with PRC and negatively with NRC (Tarakeshwar et al., 2006). As can be seen, among the correlates of religious coping are variables, understood as positive (satisfaction with life, better acceptance of illness, mental health, health-related quality of life, and positive affect) or negative (depression, anxiety, and suicide) measures of well-being. The first group of variables seems to be positively related to PRC and negatively related to NRC. In the second group, the pattern appears to be the opposite. However, this rule does not always apply. For example, in some studies PRC was found to be positively related to life satisfaction, and NRC showed no negative relationship with this variable (Abu-Raiya et al., 2015; Van Dyke et al., 2009). On the other hand, among women with breast cancer PRC was not associated with any measures of well-being, whereas NRC predicted depressive symptoms, worse overall mental health, and lower life satisfaction (Hebert et al., 2009). Moreover, if well-being is measured according to the eudaimonic approach, then we can find that NRC correlates negatively with psychological well-being, while the PRC does not correlate with it (Krok, 2015, 2017). Given that “psychological well-being is treated as an indicator of the individual's adaptation to various critical or crisis life events” (Ilska & Kołodziej-Zaleska, 2018, p. 156; Ryff, 2014, 2017), the question arises about the relationship between religious coping and mental adaptation to illness.

### Mental Adjustment to Cancer

Mental adjustment to illness is referred to as a process because in most cases the situation of illness is not uniform and changes from stage to stage (Sharpe & Curran, 2006). The aim of mental adjustment to illness is to restore mental balance despite the new and difficult situation and to eliminate unpleasant emotions (Gilbar et al., 2005). The diagnosis of cancer is followed by emotional shock, marked by disbelief and by blaming oneself and others for the disease. What usually comes later is a gradual adjustment to illness, accompanied by anxiety, which, however, does not lead to total powerlessness. In patients diagnosed with cancer, there may also be a stage of becoming independent from the disease, which consists in regaining mental balance (Dembe et al., 2019). Indeed, there are studies of young cancer patients that show their good adaptation to the disease. An American study on a sample of children with cancer (*N* = 120) revealed that they exhibited good adjustment to their illness (Williams et al., 2011). A meta-analysis of 10 studies comparing the mental adjustment of children with cancers (*N* = 676) and healthy children (*N* = 2847) revealed no differences: both groups showed equally good adjustment (Wechsler & Sánchez-Iglesias, 2013). There are also studies indicating that children with cancer show even less anxiety than their healthy peers (Phipps et al., 2001).

### Constructive and Destructive Adjustment Style and Its Relationship to Mental Health

Mental adjustment to illness manifests itself as (1) a constructive coping style, also referred to as a constructive adjustment style (CAS), characterized by motivation, the will to fight, and hope for recovery; or (2) a destructive coping style, also referred to as a destructive adjustment style (DAS), with a predominance of withdrawal behaviors, self-blame, and loss of hope (Religioni et al., 2018). In the presentation of our research, further in the text, we will analyze two aspects of CAS: (1) fighting spirit, characterized by engagement in actions intended to counter the disease, and (2) positive reevaluation, manifesting itself in noticing the positive sides of life despite the awareness of its negative sides associated with illness. DAS will also be analyzed as having two aspects: (1) helpless–hopeless, and (2) anxious preoccupation, involving the experience of fear and anxiety (Juczyński, 2001).

Research to date does not clearly show the relationship between CAS, DAS, PRC and NRC. This can only be inferred indirectly by analyzing how they are related to other variables. As it has been mentioned earlier, PRC and NRC are associated with variables related to mental health. Similarly, CAS and DAS have been studied in the context of mental health. For example, in a small sample of men with prostate cancer it was found that scores on anxious preoccupation and helpless–hopeless were positively related to anxiety and depression, whereas fighting spirit was not related to these variables (Bjorck et al., 1999). In another study a lower level of depressive symptoms was observed in women with breast and genital cancer who more often chose CAS than in those who chose DAS (Malicka et al., 2009). Amadi et al. (2016) conducted a study in two groups: people with depression and people with diabetes mellitus. In both groups it was found that fighting spirit and CAS were inversely related to depression, whereas DAS, anxious preoccupation and helpless–hopeless scales were positively related to depression. In this study NRC also correlated positively with depression whereas PRC did not correlate with it (Amadi et al., 2016).

## The Current Study

Mental adaptation to cancer appears to be linked to religious coping, although their association is not clearly defined in light of previous research. Mental adjustment to illness may also be related to support, as some studies indicate that social support is a predictor of health and coping with stress (Brewer et al., 2014; Buszman & Przybyła-Basista, 2017; Łuszczyńska & Cieślak, 2005; Schwarzer & Knoll, 2007). In this context, our study aimed to establish the relationship between religious coping (PRC and NRC) and mental adjustment to illness (CAS and DAS) among adolescent cancer patients and to determine the role of support in this relationship. We pose four hypotheses. Each of them will be preceded by a justification based on theory and/or research.

In their review, Linley and Joseph (2004) observed that PRC promoted adversarial growth, which manifested itself in the attribution of a new meaning to the difficult situation, a sense of being in control of that situation, and a positive transformation in life. These characteristics of adversarial growth also seem to be typical of CAS, which comprises a fighting spirit shown in the struggle with illness and its positive reevaluation. This makes it reasonable to indirectly infer a relationship between CAS and PRC. Similarly, Pargament’s (1996) theory itself underscores that in the process of religious coping it is essential to understand the problem and cope with it in ways that include the attribution of meaning, spiritual consolation, gaining control, and a transformation of life (Pargament et al., 2000), which seems to correspond to a positive reevaluation of the difficult situation and to the desire to overcome illness. Taking this into account, we hypothesized:

### H1a

In adolescents with cancer, there is a positive relationship between PRC and CAS (fighting spirit and positive reevaluation).

A high level of NRC, by contrast, is interpreted as maladaptive (Taheri-Kharameh et al., 2016; cf. Phelps et al., 2009), and therefore it can be treated as a sign of DAS. Studies on cancer patients showed that NRC predicted worse overall mental health and lower life satisfaction as well as some depressive symptoms (Hebert et al., 2009). Given that NRC manifests itself in symptoms such as spiritual tension and struggle with God (Pargament et al., 2011), it is reasonable to believe that individuals who use NRC build a negative image of God. It is also known that the relationship between religiosity and self-esteem is positive only in those who believe in a loving, accepting, and caring God, whereas an image of an absent, punishing, and vindictive God is accompanied by lower self-esteem (Grom, 2019). This means NRC is probably related to lowered self-esteem as well, and the lower the self-esteem, the more strongly the person experiences helplessness and hopelessness (Forward, 1992), which are typical of DAS. In this context, we posed the following hypothesis:

### H1b

In adolescents with cancer, there is a positive relationship between NRC and DAS (anxious preoccupation and helpless–hopeless).

According to Koenig (1994), faith is a more common phenomenon among people who are in socially disadvantageous situations, such as Black people, seniors, women, and individuals suffering from chronic diseases. It can therefore be expected that patients with cancers classified as chronic diseases will seek help by resorting to faith in a good God and, consequently, will exhibit a level of PRC higher than NRC. This is consistent with the results of Sabanciogullari and Yilmaz (2021): the cancer patients during chemotherapy treatment (*N* = 133) were high in PRC and low in NRC (although no relevant statistical comparisons have been made). Analyzing various studies, Phelps et al. (2009) observed that NRC was rarely found in medical samples. Therefore, our next hypothesis was as follows:

### H2a

In adolescents with cancer, the level of PRC is higher than that of NRC.

Cancers can be divided into two groups, referred to as “hormone-dependent” (e.g., breast cancer, genital cancer) and “diet-dependent” (e.g., colorectal, pancreatic, or gastric cancer). The research conducted by Juczyński (2001) revealed that CAS was predominant in the former group of cancers and DAS was predominant in the latter group. The predominant cancers in children and adolescents aged 0–19 years include leukemias, lymphomas, and brain tumors (Siegel et al., 2021), which are cancers from the former group. It could therefore be expected that in our sample of adolescents the predominant style of adjustment would be CAS. Moreover, a study of a very large sample of Polish cancer patients (*N* = 1187) suggests that their dominant strategies for coping with stress were those that make up CAS (Czerw et al., 2021). The fact that the researchers did not present a statistical analysis comparing the intensity of CAS and DAS justifies the need to verify the following hypothesis:

### H2b

In adolescents with cancer, the level of CAS is higher than that of DAS.

Further hypotheses (H3a and H3b) concern the comparison between women and men. H3a refers to PRC and NRC. In their study among Polish students (*N* = 115), Wnuk and Marcinkowski (2010) found that female students scored significantly higher on PRC than male students. Similar results were observed among adolescents (Talik, 2013). A study conducted among Iranian university students (*N* = 185) showed that, compared to men, women more often engaged in religious practices and reevaluated their difficult situation (benevolent reappraisal), believing that this was God’s will (Aflakseir & Coleman, 2011). Global statistics provided by Pew Research Center (2016) revealed that religion was more important to women than it was to men and that women more often prayed every day and attended Mass regularly, which suggests that they attach greater importance to God and religion than men do. Moreover, among cancer patients, it was more often women than men who described themselves as religious or spiritual (Rassoulian et al., 2021). Hebert et al.’s (2009) study included 198 women with stage I or II breast cancer and 86 women with stage IV breast cancer. It was found that 76% of women used PRC (i.e., partnering with God or looking to God for strength, support, or guidance) *a moderate amount* or *a lot*. NRC was much less prevalent: 15% of women reported feeling abandoned by or angry at God at least *a little*. A different study of women with breast cancer (*N* = 224) revealed a two times higher level of PRC than NRC (Zamanian et al., 2015). Taking all this into account we formulated the following hypothesis:

### H3a

In young women with cancer, the level of PRC is higher and the level of NRC is lower than in men.

Hypothesis H3b refers to CAS and DAS used by women and men. Research results concerning this issue are ambiguous. On the one hand, a study conducted among patients hospitalized for diagnosed cancer (*N* = 150) showed that, compared with men, women had a higher level of DAS, marked particularly by anxious preoccupation, and a lower level of CAS (Oniszczenko & Laskowska, 2014). On the other hand, research on patients with laryngeal cancer (*N* = 92) indicated that women scored higher on CAS than men, while men scored higher on DAS than women (Humeniuk et al., 2016). A similar result in a group of cancer patients (*N* = 150) was reported by Michałowska-Wieczorek (2006): in comparison to men, women had a higher level of CAS, marked particularly by fighting spirit, and a lower level of DAS. Additionally, Kupcewicz et al. (2017) found that women with gynecological malignancy (*N* = 102) used CAS to a greater extent than DAS. The ambiguity of the existing research points to the need for testing these relationships again, on a sample of adolescents suffering from cancers. Our hypothesis, formulated based on the most frequently recurring results, was as follows:

### H3b

In young women with cancer, the level of CAS is higher and the level of DAS is lower than in men.

In H1a, we hypothesized that there was a positive relationship between PRC and CAS, with fighting spirit as one of its aspects. A relationship between PRC and fighting spirit can be inferred, for example, from the fact that one of the key functions of religion in stressful situations consists in gaining control (Pargament et al., 2000), which is also typical of fighting spirit. In H4a, we additionally hypothesized that the relationship between PRC and fighting spirit would increase at a high level of received social support. According to what is known as the social support buffer hypothesis, a person in a difficult situation (such as being ill with cancer) who is aware of support from others reevaluates his or her skills. The person’s self-efficacy increases and their attitude to the difficult situation changes, which is conducive to better stress coping (Schwarzer & Knoll, 2007; Sęk & Cieślak, 2004). Consequently, he or she may activate an attitude of fighting spirit to overcome the disease. Therefore, we hypothesized the following:

### H4a

The relationship between PRC and fighting spirit (an aspect of CAS) is moderated by received social support. This relationship will be stronger at a high level of support.

As it has been mentioned earlier, well-being can be treated as an indicator of the individual's adaptation to critical or crisis life events (Ilska & Kołodziej-Zaleska, 2018; Ryff, 2014, 2017). The existing studies on the relationship between PRC and well-being in cancer patients have yielded diverse results. In their study on Greek cancer patients (*N* = 86), Kaliampos and Roussi (2017) found that religious coping during chemotherapy was a predictor of positive affect (a measure of subjective well-being) seven months later. Other research including patients with advanced cancer (*N* = 170) also showed that PRC was associated with better health-related quality of life and NRC with lower quality of life (Tarakeshwar et al., 2006). Contrary to this, Hebert et al. (2009) found that among women with breast cancer PRC was not associated with any measures of well-being, while NRC predicted worse mental health and life satisfaction in this group. The ambiguous research results may suggest that the strength of the relationship is influenced by a moderator. At the same time it is known that support is a predictor of well-being (Buszman & Przybyła-Basista, 2017; Łuszczyńska & Cieślak, 2005). Moreover, a study on a British Christian sample (*N* = 256) revealed that religious social support and religious coping positively predicted measures of well-being, such as self-reported current health status, health outlook, and the belief that one is more resistant to ill health (Brewer et al., 2014). We therefore hypothesized the following:

### H4b

The relationship between PRC and well-being is moderated by received social support. This relationship will be stronger at high support.

## Methods

### Respondents and Procedure

We used purposeful sampling in our study, with age from 14 to 19 years and having a cancer as the criteria. Using G-Power, it was estimated a priori that if the effect was medium *f*^2^ = 0.15, a sample of 55 people would be sufficient for the test to have a power of 0.80 in the planned moderation analysis. Assuming that the obtained effect size will be smaller (*f*^2^ = 0.10) and statistical power of test will be 0.80, the minimum total sample size was established at 81 participants. Our sample included 88 cancer patients, 44 women and 44 men (*M*_*age*_ = 17.64, *SD* = 1.68). The subjects came from all regions of Poland; they lived in villages (45%), cities with a population below 50,000 (17%), cities between 50,000 and 100,000 inhabitants (14%), and cities with more than 100,000 inhabitants (24%). Data concerning the types of cancer found in the patients, illness duration, patients’ current health situation, and their self-rated health status are presented in Table 1.

**Table 1:** Participant characteristics: numerical and percentage distribution of data concerning cancer illness and self-rated health status

	*N*	%
Type of cancer		
Leukemia	38	43.3
Lymphoma	30	34.2
Renal cancer	1	1.1
Glioma	3	3.4
Testicular cancer	1	1.1
Sarcoma	12	13.6
Medulloblastoma	1	1.1
Nasopharyngeal cancer	1	1.1
Skin cancer	1	1.1
Illness duration		
Less than one year	35	39.8
1–2 years	29	33.0
3–4 years	14	15.9
5–6 years	5	5.7
7–8 years	2	2.3
9–10 years	0	0.0
More than 10 years	3	3.3
Current health situation		
Chemotherapy	26	29.5
Radiotherapy	1	1.1
Remission	52	59.1
Observation	2	2.3
Transplant	5	5.7
Gene and stem-cell therapies	2	2.3
Self-rated health status		
Very good	28	31.8
Good	39	44.3
Moderate	18	20.5
Bad	2	2.3
Very bad	1	1.1

The research was conducted from September 2021 to April 2022, during the COVID-19 epidemic in Poland. Therefore, data were collected through an online survey; links to the survey were posted on social networks (Facebook, Instagram). Additionally, we emailed information about the research to foundations for cancer patients and contacted such patients directly using a Facebook message. The respondents were informed that their participation was anonymous and that they were free to opt out at any stage of the research. Next, we presented them with instructions and a request for consent to participate. In the case of underage individuals, consent was given by the legal guardian. The subjects could contact the researcher in writing and ask questions in case of doubts or discomfort. The procedure was approved by the Research Ethics Committee at the first author's university.

### Measures

#### The Mini-Mental Adjustment to Cancer Scale (Mini-MAC)

This 29-item questionnaire was developed by Watson et al. (1988). In the present study, we used the Polish adaptation of the measure (Juczyński, 2001). It can be used to assess patients’ reaction to a diagnosis of cancer or to determine the changes resulting from treatment and rehabilitation. Respondents rate the items on a 4-point Likert scale (1 = *definitely does not apply to me*, 2 = *does not apply to me,* 3 = *applies to me,* 4 = *definitely applies to me*). The Mini-MAC consists of four subscales: (1) Anxious Preoccupation, which measures the extent to which the changes appearing in the organism cause the patient’s anxiety and are perceived as a threat to and an uncontrollable deterioration of their health; (2) Fighting Spirit, which is meant to determine whether the patient perceives their illness as a challenge they must face and whether they take action to fight it; (3) Helpless–Hopeless, which provides information about the sense of being powerless and having no way out, experienced by the person suffering from cancer; (4) Positive Reevaluation, which concerns the ability to see hope and the positive sides of life despite cancer. Each subscale consists of 7 items. The Anxious Preoccupation and Helpless–Hopeless subscales make up the passive style of coping with illness, also referred to as the DAS, while the Positive Reevaluation and Fighting Spirit subscales make up the active coping style, also known as the CAS. Internal consistency of the Mini-MAC as determined in this study is shown in Table 2.

**Table 2:** Pearson's *r* correlations among measured variables and internal consistency

	PRC	NRC	FS	PR	AP	HH	CAS	DAS	WB	SP
PRC	–									
NRC	0.33**	–								
FS	0.19	− 0.12	–							
PR	0.65**	0.17	0.41**	–						
AP	0.21	0.46**	− 0.33**	0.16	–					
HH	0.10	0.37**	− 0.61**	− 0.21	0.61**	–				
CAS	0.49**	0.03	0.85**	0.83**	− 0.11	− 0.49**	–			
DAS	0.17	0.46**	− 0.52**	− 0.02	0.90**	− 0.89**	− 0.33**	–		
WB	0.15	− 0.08	0.54**	0.33**	− 0.39**	− 0.71**	0.52**	− 0.61**	–	
SP	0.11	− 0.22*	0.31**	0.17	− 0.26*	− 0.30**	0.29**	− 0.31**	0.37**	–
Alpha	0.95	0.82	0.77	0.62	0.84	0.89	0.77	0.91		

PRC positive religious coping, NRC negative religious coping, FS fighting spirit, PR positive reevaluation, AP anxious preoccupation, HH helpless-hopeless, CAS constructive adjustment style, DAS destructive adjustment style, WB well-being, SP support. *p < 0.05, **p < 0.01

#### The Brief Religious Coping Scale (Brief RCOPE)

This measure was developed by Pargament et al. (1998). In our study, we used the Polish adaptation of its short version (Jarosz, 2011). The measure consists of 14 items and enables the assessment of PRC and NRC as used by adolescents and adults in difficult situations. Respondents rate the items using a 4-point Likert scale: 1 = *not at all (never)*, 2 = *a little bit (seldom)*, 3 = *quite a bit (often)*, 4 = *a great deal (very often)*. The Brief RCOPE has two subscales: (1) Positive Religious Coping (PRC), composed of the first seven items, which concern issues such as seeking a relationship with God, seeking His love, care, and help, and pursuing one’s plans in accordance with God’s will; (2) Negative Religious Coping (NRC), composed of the remaining seven items, which concern issues such as questioning God’s love, challenging Divine power, and doubting God’s support and presence. The higher the score on the PRC subscale, the more effectively the respondent uses their religiosity to face up to difficult situations. The higher the score on the NRC subscale, the less effectively they use their religiosity in coping with stress (Pargament et al., 2000). Internal consistency of the Brief RCOPE as determined in this study is shown in Table 2.

#### Rating Scales

Two rating scales were used in the study. The first was used to measure the support received, and the second to measure overall well-being. Accordingly, two questions were asked: (1) How would you rate the support you get from your immediate environment?; (2) How would you rate your well-being overall? Respondents answered them using a 5-point scale (1 = *very poor*; 2 = *poor*; 3 = *average*; 4 = *good*; 5 = *very good*).

## Results

Prior to the main analyses, we calculated descriptive statistics and tested the assumptions of normality. Using the Shapiro–Wilk test, we found that only the scores on the Anxious Preoccupation, Positive Reevaluation, CAS and DAS met the normality assumptions. Therefore, in the next step, we analyzed skewness. PRC and NRC, Anxious Preoccupation, Helpless–Hopeless, and DAS were slightly positively skewed (from 0.20 to 0.88), while Fighting Spirit, Positive Reevaluation, and CAS were slightly negatively skewed (from − 0.26 to − 0.42). All skewness coefficients were in the − 1 to 1 range, which means skewness was not strong enough to require further attention and could be ignored (George & Mallery, 2010). Next, we computed Pearson bivariate correlations for all variables measured in the study (Table 2).

We found significant positive correlations of PRC with CAS and Positive Reevaluation. The correlation between PRC and Fighting Spirit appeared to be non-significant. This means that H1a was only partly supported. We also found other significant positive correlations of NRC with DAS and its aspects—Anxious Preoccupation and Helpless–Hopeless. This means H1b was fully confirmed.

To test H2a and H2b, we used Student’s *t* test for dependent samples (Table 3). We found that PRC was used to a greater extent than NRC in the current sample, which means H2a was supported, though the effect size was small (*g* = 0.38). H2b was also supported, because the scores for DAS proved to be significantly lower than for CAS. In this case, however, the effect size was large (*g* = 1.12). To conclude, adolescents with cancer more intensively use their religiosity positively than negatively to cope with the illness. Additionally, they show a constructive rather than destructive adjustment to cancer.

**Table 3:** Comparison of adolescents on positive versus negative religious coping and constructive versus destructive adjustment styles

	*M*	*SD*	*t*	*df*	*p*	*g*
Positive religious coping	14.59	6.14	3.62	87	< 0.001	0.38
Negative religious coping	12.14	4.61				
Constructive adjustment style	41.74	6.46	10.57	87	< 0.001	1.12
Destructive adjustment style	27.99	8.45				

To test H3a and H3b, we used Student’s *t*-test for independent samples (Table 4). We hypothesized (H3a) that in young women with cancer the level of PRC is higher and the level of NRC is lower than in men. However, the analyses did not yield statistically significant differences between the groups, which means H3a was not supported.

**Table 4:** Comparison of men and women on positive and negative religious coping and constructive and destructive adjustment styles

	Sex	*M*	*SD*	*t*	*df*	*p*	*g*
Positive religious coping	F	14.75	6.28	0.24	86	0.809	0.05
	M	14.43	6.05				
Negative religious coping	F	12.30	4.44	0.32	86	0.748	0.07
	M	11.98	4.81				
Constructive adjustment style	F	42.89	6.02	1.68	86	0.048	0.36
	M	40.59	6.75				
Destructive adjustment style	F	29.23	8.12	1.38	86	0.085	0.29
	M	26.75	8.67				

F female, M male

According to H3b, in young women with cancer the level of CAS is higher and the level of DAS is lower than in men. H3b was only partly supported; we found that women used CAS to a greater extent than men. At the same time, they showed a tendency to use DAS more intensively than men.

The last hypotheses (H4a and H4b) were verified using moderation analyses (Table 5), which we performed using the PROCESS macro (Model 1; Hayes, 2018). In order to avoid Type II errors, the bootstrapped samples in the PROCESS macro were set to 5000 with 95% bias-corrected confidence intervals.

**Table 5:** The moderations analyses for positive religious coping as an independent variable, the support as a moderator, and fighting spirit or well-being as dependent variables

Dependent variable	*R*^*2*^	B	*t*	Interaction					
				Effect_L_	95%CI_L_	Effect_M_	95% CI_M_	Effect_H_	95% CI_H_
Fighting spirit	.169	.200	2.26*	− .003	[− .160; .154]	− .003	[− .160; .154]	**.197**	**[.043; .351]**
Well-being	.210	.051	2.50*	− .010	[− .046; .027]	− .010	[− .046; .027]	**.042**	**[.006; .078]**

The significant effects are highlighted in bold

*R*^2^ = amount of variance explained by the model; CI = confidence interval; Effect_L,M,H_ = conditional effects of the predictor at values of the low (16th percentile), medium (50th percentile), and high (84th percentile) moderator, respectively

*p < .05

It was hypothesized that the relationship between PRC and fighting spirit (H4a) and the relationship between PRC and well-being (H4b) were moderated by received social support. We expected that these relationships would be stronger at a high level of support. As proposed in the PROCESS macro, the conditional effects of the predictor (PRC) were tested at three levels of the moderator—namely, at low (16th percentile), average (50th percentile), and high (84th percentile) received support scores. It turned out that the link between PRC and fighting spirit (H4a) was not significant in subjects with low and medium levels of received support, but became statistically significant in those with a high level of support. A similar pattern was observed for the relationship between PRC and well-being. Thus, hypotheses H4a and H4b were both confirmed.

## Discussion

The study aimed to determine the role that religious coping played in adolescents in the context of mental adjustment to cancer. Additionally, we investigated the function of support received during illness.

### Positive Religious Coping and Reevaluation of Illness

H1a postulated that among adolescents suffering from cancer there was a positive relationship between PRC and CAS (fighting spirit and positive reevaluation). In the end, H1a was only partly supported. We found a positive relationship between PRC and one aspect of CAS: positive reevaluation. According to Pargament’s (1997) theory, religious coping aims to transform meaning if maintaining it proves to be impossible in a difficult situation. As the use of PRC is associated with the experience of secure attachment to the sacred and with adopting a benevolent world view, it becomes understandable that the transformation of meaning often leads to a positive reevaluation of one’s life or the illness situation. As regards fighting spirit, which is the other component of CAS, a positive correlation with PRC was not confirmed. This result is worth highlighting. Trusting in God and seeing Him as a support does not encourage people to fight cancer, but promotes the search for a new positive meaning of the disease. The relationship between PRC and fighting spirit manifested itself only in the context of received support, which will be discussed further, in the passage devoted to H4a.

### Negative Religious Coping and Anxiety, Helplessness and Hopelessness

We also hypothesized (H1b) that among adolescents with cancer there was a positive relationship between NRC and DAS (anxious preoccupation and helpless–hopeless). H1b was supported. This is consistent with a study, showing that NRC is related to negative affect and psychological distress (depression, anxiety, somatization) in urban early adolescents (Van Dyke et al., 2009). Similarly, Hebert et al. (2009) found that NRC predicted worse overall mental health, lower life satisfaction and increase in depressive symptoms in women with breast cancer. Also Amadi et al. (2016) showed that the choice of NRC contributed to depressive symptoms. Given that depression is understood as mood deterioration, lowered activity, and decreased experience of pleasure (ICD-10, 2008), it can be assumed that the use of NRC may be accompanied by feelings of helplessness and hopelessness and lead to anxious preoccupation (DAS).

It seems that self-esteem may play an important role in this relationship. It turns out that a person fighting against God and other people (NRC) (Pargament et al., 2011) usually has low self-esteem (Grom, 2019). In a difficult situation such as a dangerous disease, he/she does not find the strength to fight the threat. In addition, conflicted with God and the world – he/she also does not find this strength outside. As a result, they may experience fear, hopelessness and helplessness (Forward, 1992). Of course, confirmation of this mechanism requires further research.

### Predominant Type of Religious Coping and Mental Adjustment to Cancer Among Adolescents

H2a, in which we hypothesized that young people with cancers were higher in PRC than in NRC, was fully supported. This is consistent with the other findings. For example, Sabanciogullari and Yilmaz (2021) found that cancer patients were high in PRC and low in NRC. Analogous conclusions seem to follow from a study on women with breast cancer (Hebert et al., 2009). Similar results can be observed not only in cancer patients but also in chronically ill individuals (Koenig, 1994) and in healthy teenagers (Van Dyke et al., 2009). Analyzing studies conducted on different groups of ill subjects, Phelps et al. (2009) found that NRC was seldom found in medical samples.

Additionally, we hypothesized (H2b) that in adolescents with cancer the level of CAS was higher than that of DAS. We found support for this hypothesis too. This result is in line with the findings by Czerw et al. (2021). Their study on a large sample of Polish cancer patients seemed to show that CAS strategies, that is, fighting spirit and positive reevaluation prevailed (although the researchers did not provide a statistical analysis comparing the intensity of CAS and DAS). Our analyses confirmed that among adolescents with cancer the level of CAS was higher than DAS. This predominance of CAS over DAS may explain the good adaptation to the disease among young cancer patients (Wechsler & Sánchez-Iglesias, 2013; Williams et al., 2011). In this context, good adaptation to cancer would stem from the will to fight and overcome the disease (fighting spirit) while at the same time appreciating life and noticing its positive aspects (positive reevaluation).

Considering H2a and H2b, it is worth noting that even if previous studies have suggested conclusions consistent with our results, studies of oncology patients have so far lacked adequate statistical comparative analyzes in this regard. All the more, there were no such data for adolescents with cancer. In this sense, our study contributes to the existing knowledge.

### Gender Differences in the Use of Religious Coping Strategies

Hypothesis H3 concerned gender differences in the choice of coping strategies among adolescents with cancer. In H3a we hypothesized that in young women with cancer the level of PRC was higher and the level of NRC was lower than in men. This hypothesis was not supported, as we found no statistically significant gender differences in the use of PRC and NRC. It is worth emphasizing that we posed our hypothesis by referring to the results of analogous comparisons in groups of healthy adolescents and by inferring from other studies of cancer patients and healthy adults, which, however, did not explicitly address such a comparison. We knew from research conducted among university students (Wnuk & Marcinkowski, 2010) and adolescents (Talik, 2013) that members of the female sex scored higher on PRC than those of the male sex. Other studies showed that women are a more religious sex than men (Aflakseir & Coleman, 2011), which was also confirmed in a study of cancer patients (Rassoulian et al., 2021). Additionally, some studies have shown that women with cancer use PRC to a greater extent than NRC (Hebert et al., 2009; Zamanian et al., 2015). In this context, it seems necessary to replicate our study on another sample of adolescents with cancer to see if there are significant gender differences in the use of PRC and NRC. At the same time it is worth considering if the effect of blurred gender differences that we found was not caused by the experience of the COVID-19 epidemic, which was in progress during our project. There are studies on religious coping during the pandemic that show no gender differences in the levels of PRC (Shameem et al., 2022) or NRC (Francis et al., 2021). It is conceivable that the epidemic situation intensified cancer patients’ experience of threat to their life, which may have influenced their choice of strategies for coping with stress through religion. Further questions arise, however: Did the changes concern one sex, and if so, which one? Or did the changes concern both sexes? If so, did they have opposite directions, reducing the differences between young men and women? Thus, as can be seen, the issue of gender differences in the use of religious coping strategies requires further study.

### Gender Differences in Mental Adjustment to Illness

In H3b we hypothesized that in young women with cancer the level of CAS was higher and the level of DAS was lower than in men. It should be stressed that we posed this hypothesis by referring to the results of analogous comparisons among adult cancer patients, because there were no such data for adolescents with cancer. Moreover, the existing results were ambiguous and our hypothesis was formulated based on the most frequently recurring findings. Therefore, it is not surprising that this hypothesis was only partially supported. Indeed, the women in our sample used CAS to a greater extent than men. These findings are consistent with the study of patients with laryngeal cancer (Humeniuk et al., 2016), in which it was found that women scored higher on CAS than men. We did not, however, find support for the part of H3b according to which the level of DAS in women was lower than in men. Although such a pattern was found by Humeniuk et al. (2016) and Michałowska-Wieczorek (2006), the young women in our study exhibited a tendency to use DAS to a greater extent than men. This in turn is in line with the study by Oniszczenko and Laskowska (2014), who found that, compared to men, women with cancer had a higher level of DAS, marked particularly by anxious preoccupation. Since no such comparisons have been made to date among adolescents with cancer, our study contributes to the body of knowledge. At the same time, our findings need to be replicated, given the differences in the proportion of CAS to DAS in results obtained from adult samples. These differences can be explained by the fact that adults with cancer tend to simultaneously use many strategies for coping with illness, both constructive and destructive ones (Nipp et al., 2016). Perhaps this broad spectrum of coping strategies gives the patient a sense of greater agency and control over the illness, but at the same time it may lead to results more varied within and between groups. In this context, it will be useful to test whether different samples of adolescents with cancer will also show differences in the CAS to DAS ratio.

### Social Support in the Relationship between Positive Religious Coping and Fighting Spirit

We also hypothesized (H4a) that social support received during illness would be a moderator in the relationship between PRC and fighting spirit, which is an element of CAS. Hypothesis H4a was supported. At a level of received support rated as very high, PRC turned out to promote fighting spirit. This is new knowledge and is perhaps the most important result of the conducted research. Contrary to expectations, it was found that PRC is not a sufficient condition for the appearance of a fighting spirit against illness. A positive attitude toward God, associated by Koenig (1994) with seeking support and consolation in God, is usually not enough to take up the fight against a life-threatening illness (non-significant correlation between PRC and fighting spirit in our study). Rather, PRC is conducive to a positive reevaluation of the disease (H1a), that is, treating cancer as part of God's plan and accepting its consequences. However, it turns out that very strong support from loved ones that accompanies a positive attitude toward God (PRC) can motivate one to fight cancer. It is possible that a sick person interprets the support he/she experiences from people as a manifestation of God's action. This allows him/her to fight for his/her life and at the same time feel that he/she is not opposing God's plans for himself/herself, even though illness is an element of that plan. Some studies have found that support given to a person in a difficult situation allows them to rate their skills and self-efficacy higher and to adopt a more positive attitude towards hardship, which promotes better coping with stress (Schwarzer & Knoll, 2007; Sęk & Cieślak, 2004). It is possible that this is the mechanism through which PRC and social support activate an attitude of fighting spirit to overcome the disease. This is consistent with other studies, where social support was a predictor of health status and coping with stress (Brewer et al., 2014; Buszman & Przybyła-Basista, 2017; Łuszczyńska & Cieślak, 2005).

### Social Support in the Relationship between Positive Religious Coping and Well-Being

Previous research on the relationship between PRC and the well-being of cancer patients has yielded contradictory results (Hebert et al., 2009; Kaliampos & Roussi, 2017). The inconsistent findings may suggest that the strength or direction of the relationship is influenced by a moderator. We hypothesized (H4b) that social support was a moderator of this relationship. As in the case of H4a, it turned out that the postulated relationship between PRC and well-being was significant at very high support. It seems that the mechanism through which PRC accompanied by social support promote well-being may be similar to the mechanism through which PRC and social support activate the fighting spirit to overcome illness. Our study showed that high level of PRC does not guarantee high level of well-being (no significant correlation between PRC and well-being). PRC is understood as a process in which a person, trusting in God, seeks meaning in life, greater mastery and control over his or her life, comfort from God, and closeness to God and others (Pargament et al., 2011). It is conceivable, however, that different people reach this goal of the search at different times, and for some it can be a long and difficult process. Therefore, PRC is often not enough to feel good. Strong social support can change this, as support is known to be a predictor of well-being (Buszman & Przybyła-Basista, 2017; Łuszczyńska & Cieślak, 2005). Crean (2004) found that social support was positively related to adolescents' school competence. Similarly, Schwarzer and Knoll (2007) demonstrated the facilitating effect of support on self-efficacy, which in turn promoted coping with the aftermath of major surgery. Taking all this into account, we argue that social support, by enhancing self-efficacy, can foster well-being in two ways: through the emergence of positive emotions (positive measures of well-being; Pearlin et al., 1981) and through the reduction of depressive symptoms and anxiety (negative measures of well-being; Yarchesky et al., 2001).

### Assessment of Support

It is worth noting that, in our study, when adolescents with cancer were asked to evaluate the support they received on a scale from 1 to 5, 62.5% of the sample rated this support as very good (5), 22.7% as good (4), 13.6% as average (3), and 1.1% as poor (2); no one rated it as very poor (1). At the same time, our moderation analyses show that only the evaluation of support as very good is conducive to the activation of the spirit of fighting the disease and to the increase in well-being in adolescent cancer patients using PRC. This effect is not observed, however, when support is rated as good or lower on the scale (score 4–1). Two conclusions arise in this context. Firstly, most teenagers with cancer can count on very good support from other people, which is very important for fighting the disease and the recovery process. Secondly, support rated as good in this group may in fact mean that support is insufficient—to protect their closest others, our respondents may have overrated the support they received.

## Limitations

The sample was small and heterogeneous. It consisted of patients with various types of cancer, diverse in terms of illness duration and stage of disease. For example, 59.1% of participants reported disease remission during the study. Thus, there may be some doubt as to whether the results are a good reflection of the situation of young people suffering from cancer. Therefore, it should be emphasized that none of the participants remained in remission long enough for the doctors to conclude that the illness was cured. Consequently, the situation of young people in remission was considered to be still difficult and typical of many cancer patients. Such thinking is supported by the fact that our findings are largely consistent with those of other studies. At the same time, replication of our research on young patients in the acute phase of cancer would be valuable. All the shortcomings of our sample are partly justifiable by the fact that the subjects were very young people, aged 14–19 years—a hard-to-reach group of cancer patients. Due to these weaknesses, caution would be advised in generalizing the results to other samples. This refers mainly to people with specific types of cancer or to non-adolescents, as each type of cancer and each developmental stage is unique in some sense.

Moreover, due to the epidemic, the research was conducted remotely. On the one hand, an online study guarantees greater anonymity to participants; on the other, it may have negatively affected the reliability of questionnaire completion. The pandemic situation itself may have had some effect on the results. Various kinds of restrictions introduced across the country due to COVID-19 hindered functioning in many areas. In cancer patients with decreased immunity, this may have increased a sense of threat to life and led to prolonging the isolation, negatively affecting the psyche. Another potential limitation is associated with the lack of control for the level of participants’ religiosity. The author of the Polish adaptation of the Brief RCOPE suggested that the measure should preferably be used with religious respondents (Jarosz, 2011). However, Pargament (1997), who introduced the concept of religious coping, did not link his theory to the sacred of any particular kind, which allowed us to assume that both believers and nonbelievers could activate PRC or NRC if they found themselves in a difficult situation.

Taking all these shortcomings into account, further extensive research among adolescent cancer patients is needed that would include more subjects, that would divide them not only according to age but also according to type of cancer, illness duration, and stage of disease, and that would control for their attitude to religion. Since our study was conducted on a small group of patients, the results require replication on a larger cohort. Analyses on a large sample will not only allow us to confirm our findings, but also enable to apply additional, more sophisticated statistical analyses, which were not possible here due to the small number of participants.

## Conclusions and Practical Implications

In summary, among adolescent cancer patients, PRC was associated with CAS (mainly with the aspect of positive reevaluation), while NCR was associated with DAS. Participants were higher in PRC than in NRC and were higher in CAS than in DAS. In young women, CAS was higher than in men. Finally, PRC turned out to promote fighting spirit and well-being when the level of received support was rated as very high.

The results of our study may prove to be helpful in clinical practice when helping cancer patients, especially children and adolescents. They may be useful not only to medical and psychological staff and clergy but also to patients’ family members. Our findings show that the level of patients’ adjustment to cancer can be increased not only through medical or psychotherapeutic interventions but also through conversations about the experience of illness-related anxiety, the role of religiosity in difficult situations, their image of God, and the need for support from their loved ones. Illness entails changes in the current lifestyle, but positive religious commitment and support received from others can help a person cope with the difficult situation constructively.

## References

[CR1] Abu-Raiya H, Hamama L, Fokra F (2015). Contribution of religious coping and social support to the subjective well-being of Israeli Muslim parents of children with cancer: A preliminary study. Health & Social Work.

[CR2] Aflakseir A, Coleman P (2011). Initial development of the Iranian Religious Coping Scale. Journal of Muslim Mental Health.

[CR3] Amadi KU, Uwakwe R, Odinka PC, Ndukuba AC, Muomah CR, Ohaeri JU (2016). Religion, coping and outcome in out-patients with depression or diabetes mellitus. Acta Psychiatrica Scandinavica.

[CR4] Bjorck JP, Hopp DP, Jones LW (1999). Prostate cancer and emotional functioning: Effects of mental adjustment, optimism, and appraisal. Journal of Psychosocial Oncology.

[CR5] Buszman K, Przybyła-Basista H (2017). Polska adaptacja Wielowymiarowej Skali Spostrzeganego Wsparcia Społecznego [The Polish adaptation of the Multidimensional Scale of Perceived Social Support]. Polskie Forum Psychologiczne.

[CR6] Brewer G, Robinson S, Sumra A, Tatsi E, Gire N (2014). The influence of religious coping and religious social support on health behaviour, health status and health attitudes in a British Christian sample. Journal of Religion and Health.

[CR7] Crean H (2004). Social support, conflict, major life stressors and adaptive coping strategies in Latino middle school students: An interactive model. Journal of Adolescent Research.

[CR8] Czerw A, Religioni U, Szymański F, Nieradko-Heluszko A, Mękal D, Hering D, Kowalczuk A, Merks P, Borowska M, Bogdan M, Pajewska M (2021). Normalization of the Mini-MAC (Mental Adjustment to Cancer) Questionnaire among Cancer Patients. International Journal of Environmental Research and Public Health.

[CR9] Dembe, K., Chłosta, P. L., & Czech, A. K. (2019). *Oblicza choroby nowotworowej. Poradnik dla pacjentów z nowotworami układu moczowo-płciowego oraz dla ich rodzin lub opiekunów* [The faces of cancer: A manual for genitourinary cancer patients and their families or caregivers]. Polskie Towarzystwo Urologiczne.

[CR10] Desmond SA, Morgan KH, Kikuchi G (2010). Religious development: How (and why) does religiosity change from adolescence to young adulthood?. Sociological Perspectives.

[CR11] Forward, S. (1992). *Toksyczni rodzice* [Toxic parents]. Jacek Santorski & co Agencja Wydawnicza.

[CR12] Francis B, Ken CS, Han NY, Ariffin MAA, Md Yusuf MH, Wen LJ, Petrus CF, Chin BH, Gill JS, Sulaiman AH, Said MA (2021). Religious coping during the COVID-19 pandemic: Gender, occupational and socio-economic perspectives among Malaysian frontline healthcare workers. Alpha Psychiatry.

[CR13] George, D., & Mallery, M. (2010). *SPSS for Windows Step by Step: A Simple Guide and Reference, 17.0 update*. Pearson.

[CR14] Gilbar O, Or-Han K, Plivazky N (2005). Mental adjustment, coping strategies, and psychological distress among end-stage renal disease patients. Journal of Psychosomatic Research.

[CR15] Grom, B. (2019). *Psychologia religii* [Psychology of religion]*.* Wydawnictwo Naukowe PWN.

[CR16] Hayes AF (2018). Introduction to mediation, moderation, and conditional process analysis: A regression-based approach.

[CR17] Hebert R, Zdaniuk B, Schulz R, Scheier M (2009). Positive and negative religious coping and well-being in women with breast cancer. Journal of Palliative Medicine.

[CR18] Humeniuk E, Wolańska K, Tarkowski Z (2016). Czynniki wpływające na przystosowanie psychiczne do choroby pacjentów po laryngektomii [Factors influencing mental adjustment to illness in patients after laryngectomy]. Otorynolaryngologia.

[CR19] ICD-10 (2018). Międzynarodowa statystyczna klasyfikacja chorób i problemów zdrowotnych [International statistical classification of diseases and health problems]. Centrum systemów informacyjnych i ochrony zdrowia 2012.

[CR20] Ilska M, Kołodziej-Zaleska A (2018). Dobrostan hedonistyczny i eudajmonistyczny w sytuacjach kryzysów normatywnych i nienormatywnych [Hedonic and eudaimonic well-being in situations of normative and non-normative crises]. Zeszyty Naukowe Politechniki Śląskiej.

[CR21] Jarosz M, Jarosz M (2011). Skala Religijnego Radzenia Sobie—wersja skrócona (Brief RCOPE) [The Brief Religious Coping Scale—Short version (Brief RCOPE)]. Psychologiczny pomiar religijności [Psychological measurement of religiosity].

[CR22] Juczyński Z, Szustrowa T (2001). Skala Przystosowania Psychicznego do Choroby Nowotworowej—Mini MAC [The Mental Adjustment to Cancer Scale—Mini-MAC]. Narzędzia pomiaru w promocji i psychologii zdrowia [Measures in health promotion and psychology].

[CR23] Kaliampos A, Roussi P (2017). Religious beliefs, coping, and psychological well-being among Greek cancer patients. Journal of Health Psychology.

[CR24] Koenig, H. G. (1994). Religion and hope for the disabled elder. In J. Levin (red.), *Religion in aging and health. Theoretical foundations and methodological frontiers* (pp. 18–51). Sage Publications.

[CR25] Krok D (2015). The role of meaning in life within the relations of religious coping and psychological well-being. Journal of Religion and Health.

[CR26] Krok, D. (2017). *W poszukiwaniu znaczenia choroby nowotworowej* [In search of the meaning of cancer]. Wydawnictwo Uniwersytetu Opolskiego.

[CR27] Kupcewicz E, Olewińska J, Pikus H, Jóźwik M (2017). Mental adaptation to cancer in women with gynaecological cancer. Journal of Pre-Clinical and Clinical Research.

[CR28] Levenson MR, Aldwin CM, Igarashi H, Paloutzian RF, Park CL (2013). Religious development from adolescence to middle adulthood. Handbook of the psychology of religion and spirituality.

[CR29] Linley PA, Joseph S (2004). Positive change following trauma and adversity: A review. Journal of Traumatic Stress.

[CR30] Łuszczyńska A, Cieślak R (2005). Protective, promotive, and buffering effects of perceived social support in managerial stress: The moderating role of social support. Anxiety, Stress and Coping: An International Journal.

[CR31] Malicka I, Szczepańska J, Anioł K, Rymaszewska J, Woźniewski M (2009). Zaburzenia nastroju i strategie przystosowania do choroby u kobiet leczonych operacyjnie z powodu nowotworu piersi i narządów rodnych [Mood disorders and strategies of adjustment to illness in women surgically treated for breast and genital cancer]. Współczesna Onkologia.

[CR32] Michałowska-Wieczorek I (2006). Rola wsparcia w zmaganiu się z chorobą nowotworową [The role of support in coping with neoplastic disease]. Psychoonkologia.

[CR33] Mueller PS, Plevak DJ, Rummans TA (2001). Religious involvement, spirituality, and medicine: Implications for clinical practice. Mayo Clinic Proceedings.

[CR34] Nipp RD, El-Jawahri A, Fishbein JN, Eusebio J, Stagl JM, Gallagher ER, Park ER, Jackson VA, Pirl WF, Greer JA, Temel JS (2016). The relationship between coping strategies, quality of life, and mood in patients with incurable cancer. American Cancer Society.

[CR35] Oniszczenko W, Laskowska A (2014). Emotional reactivity, coping style and cancer trauma symptoms. Archives of Medical Science.

[CR36] Pargament KI, Shafranske EP (1996). Religious methods of coping: Resources for the conservation and transformation of significance. Religion and the clinical practice of psychology.

[CR37] Pargament KI (1997). The psychology of religion and coping: Theory, research, practice.

[CR38] Pargament KI, Smith BW, Koenig HG, Perez L (1998). Patterns of positive and negative religious coping with major life stressors. Journal for the Scientific Study of Religion.

[CR39] Pargament KI, Koenig HG, Perez LM (2000). The many methods of religious coping: Development and initial validation of RCOPE. Journal of Clinical Psychology.

[CR40] Pargament K, Feuille M, Burdzy D (2011). The Brief RCOPE: Current psychometric status of a Short Measure of Religious Coping. Religions.

[CR41] Pearlin LI, Menaghan EG, Lieberman MA, Mullan JT (1981). The stress process. Journal of Health and Social Behavior.

[CR42] Pew Research Center. (2016). The gender gap in religion around the world. Downloaded 14.09.2022 from https://www.pewresearch.org/religion/2016/03/22/the-gender-gap-in-religion-around-the-world/

[CR43] Phelps AC, Maciejewski PK, Nilsson M, Balboni TA, Wright AA, Paulk ME, Trice E, Schrag D, Peteet JR, Block SD, Prigerson HG (2009). Religious coping and use of intensive life-prolonging care near death in patients with advanced cancer. Journal of the American Medical Association.

[CR44] Phipps S, Steele RG, Hall K, Leigh L (2001). Repressive adaptation in children with cancer: A replication and extension. Health Psychology.

[CR45] Puchalska-Wasyl MM, Łysiak M, Zarzycka B (2022). Deconversion and identity formation in adolescents: The role of internal dialogues, gender, and religiousness of parents. International Journal for the Psychology of Religion.

[CR46] Rassoulian A, Gaiger A, Loeffler-Stastka H (2021). Gender differences in psychosocial, religious, and spiritual aspects in coping: A cross-sectional study with cancer patients. Women’s Health Reports.

[CR47] Religioni U, Czerw A, Deptała A (2018). Patient mental adjustment to selected types of cancer. Psychiatria Polska.

[CR48] Ryff CD (2014). Psychological well-being revisited: Advances in science and practice. Psychotherapy and Psychosomatic.

[CR49] Ryff CD (2017). Eudaimonic well-being, inequality, and health: Recent findings and future directions. International Review of Economic.

[CR50] Sabanciogullari S, Yilmaz FT (2021). The effect of religious coping on hope level of cancer patients receiving chemotherapy. Journal of Religion and Health.

[CR51] Schwarzer R, Knoll N (2007). Functional roles of social support within the stress and coping process: A theoretical and empirical overview. International Journal of Psychology.

[CR52] Sęk H, Cieślak R, Sęk H, Cieślak R (2004). Wsparcie społeczne – sposoby definiowania, rodzaje i źródła wsparcia, wybrane koncepcje teoretyczne [Social support: Definitions, types, and sources of support, selected theories]. Wsparcie społeczne, stres i zdrowie [Social support, stress, and health].

[CR53] Shameem F, Mahnoor A, Mamoona M (2022). Religious coping and young adult’s mental well-being during Covid-19: Testing a double moderated mediation model. Archive for the Psychology of Religion.

[CR54] Sharpe L, Curran L (2006). Understanding the process of adjustment to illness. Social Science and Medicine.

[CR55] Siegel RL, Miller KD, Fuchs HE, Jemal A (2021). Cancer statistics. A Cancer Journal for Clinicians.

[CR56] Sung H, Ferlay J, Siegel RL, Laversanne M, Soerjomataram I, Jemal A, Bray F (2021). Global cancer statistics 2020: GLOBOCAN estimates of incidence and mortality worldwide for 36 cancers in 185 countries. A Cancer Journal for Clinicians.

[CR57] Taheri-Kharameh Z, Zamanian H, Montazeri A, Asgarian A, Esbiri R (2016). Negative religious coping, positive religious coping, and quality of life among hemodialysis patients. Nephro-Urology Monthly.

[CR58] Talik, E. (2013). *Jak trwoga to do Boga? Psychologiczna analiza religijnego radzenia sobie ze stresem u młodzieży w okresie dorastania* [When in fear, God is near: A psychological analysis of religious coping with stress in adolescents]. Gdańskie Wydawnictwo Psychologiczne.

[CR59] Tarakeshwar N, Vanderwerker LC, Paulk E, Pearce MJ, Kasl SV, Prigerson HG (2006). Religious coping is associated with the quality of life of patients with advanced cancer. Journal of Palliative Medicine.

[CR60] Van Dyke CJ, Glenwick DS, Cecero JJ, Kim SK (2009). The relationship of religious coping and spirituality to adjustment and psychological distress in urban early adolescents. Mental Health, Religion and Culture.

[CR100] Watson M, Greer S, Young J, Inayat Q, Burgess C, Robertson B (1988). Development of a questionnaire measure of adjustment to cancer: The MAC scale. Psychological Medicine.

[CR61] Wechsler AM, Sánchez-Iglesias I (2013). Psychological adjustment of children with cancer as compared with healthy children: A meta-analysis. European Journal of Cancer Care.

[CR62] Williams NA, Allen MT, Phipps S (2011). Adaptive style and physiological reactivity during a laboratory stress paradigm in children with cancer and healthy controls. Journal of Behavioral Medicine.

[CR63] Wnuk M, Marcinkowski JT (2010). Religijne korelaty jakości życia młodzieży studiującej – moderacyjna rola płci [Religious correlates of quality of life in university students: The moderating role of gender]. Problemy Higieny i Epidemiologii.

[CR64] Yarchesky A, Mahon N, Yarchesky T (2001). Social support and well-being in early adolescence. Clinical Nursing Research.

[CR65] Zamanian H, Eftekhar-Ardebili H, Eftekhar-Ardebili M, Shojaeizadeh D, Nedjat S, Taheri-Kharameh Z, Daryaafzoon M (2015). Religious coping and quality of life in women with breast cancer. Asian Pacific Journal of Cancer Prevention.

[CR66] Zarzycka B, Puchalska-Wasyl MM, Łysiak M (2022). Deconversion processes and quality of life among Polish adolescents: The mediating role of social support. International Journal for the Psychology of Religion.

